# Targeting IL-6 Is a Potential Treatment for Primary Cystic Craniopharyngioma

**DOI:** 10.3389/fonc.2019.00791

**Published:** 2019-08-21

**Authors:** Sydney Grob, David M. Mirsky, Andrew M. Donson, Nathan Dahl, Nicholas K. Foreman, Lindsey M. Hoffman, Todd C. Hankinson, Jean M. Mulcahy Levy

**Affiliations:** ^1^Department of Pediatrics, University of Colorado Denver, Aurora, CO, United States; ^2^The Morgan Adams Foundation Pediatric Brain Tumor Research Program, Children's Hospital Colorado, Aurora, CO, United States; ^3^Department of Radiology, University of Colorado Denver, Aurora, CO, United States; ^4^Department of Neurosurgery, Children's Hospital Colorado and University of Colorado—Anschutz Medical Campus, Aurora, CO, United States; ^5^Department of Pharmacology, University of Colorado Denver, Aurora, CO, United States

**Keywords:** craniopharyngioma, IL-6, tocilizumab, pediatric, bevacizumab

## Abstract

Adamantinomatous craniopharyngioma (ACP) makes up between 6 and 8% of pediatric brain tumors and is the most common pediatric tumor arising in the sellar/suprasellar region of the brain. The 10-year survival for patients diagnosed with craniopharyngioma ranges between 64 and 92%, but complicating factors such as location, common cyst formation, and potential hypothalamic infiltration cause significant morbidity in this population. There are a number of therapeutic options for children with ACP, including surgery, radiation, and cyst directed therapies such as interferon and bleomycin. Research has raised concerns regarding the efficacy and side effects associated with these conventional therapies, as well as with the difficulty in treating recurrent cystic ACP. Evidence from our group and others has shown that the cystic and solid tumor components of craniopharyngioma have high levels of IL-6R and IL-6, providing a potential target for therapy. Tocilizumab, a humanized monoclonal antibody, acts against soluble and membrane bound IL-6R, and has been widely utilized in pediatric patients. Two patients with recurrent cystic ACP were offered systemically administered tocilizumab or a combination of tocilizumab and bevacizumab on a compassionate use basis. Both patients' tumors had a significant response, with decreased cyst burden, supporting the assertion that tocilizumab with or without bevacizumab may be an option for patients suffering from cystic ACP.

## Introduction

Adamantinomatous craniopharyngioma (ACP) is a neurologically devastating chronic disease with morbidity that far outweighs the mortality risk. It comprises 6–8% of pediatric brain tumors (1, 2) and is the most common tumor of the sellar/suprasellar region in children (3). ACP is among a growing number of pediatric brain tumors in which tumor growth can often be controlled with 10-year survival ranging between 64 and 92% (4) however, ACP's suprasellar location and propensity for cyst formation [present in ~90% (5)] and hypothalamic infiltration predispose many children to a life of severe disability. As such, ACP has been associated with the lowest quality of life scores of any pediatric brain tumor (6).

Therapeutic modalities for children with ACP include maximally-safe surgery, radiation, and cyst-directed therapies, such as interferon and bleomycin. None of these approaches, however, are directed against unique biological characteristics of ACP. Furthermore, after many years of use, each has been shown to be associated with shortcomings regarding clinical efficacy and/or side effects (7–11) especially when observed within the framework of ACP as a chronic disease.

Basic science research in ACP has demonstrated a single recurrent somatic mutation of the *CTNNB1* gene (12, 13), which leads to WNT pathway activation. This mutation is, unfortunately, not currently therapeutically targetable and is present in a minority of ACP cells (14), suggesting additional pathogenic drivers. Work using recently developed genetically engineered mouse models (GEMMs) of ACP indicates that the tumor arises from precursors of the anterior pituitary gland or Rathke's Pouch (15) hence, ACP may not reside within an immunoprivileged location behind the blood-brain barrier [BBB]. Further work suggests that a unique paracrine mechanism drives pathological tumor behavior by cells that lack the *CTNNB1* mutation (14, 15). This assertion is supported by work describing both the pediatric ACP transcriptome (16) and inflammatory milieu (17), which indicates a proinflammatory environment in ACP tissue and cyst fluid. These studies demonstrated highly upregulated levels of IL-6R and IL-6 in cyst fluid and solid tumor tissue. While the precise mechanism of paracrine signaling is not yet known, IL-6/IL-6R blockade may hold therapeutic relevance for ACP.

Tocilizumab, a humanized monoclonal antibody against soluble and membrane bound IL-6R, is approved by the U.S. Food and Drug Administration for systemic administration in pediatric patients age >2 years. Indications include the treatment of systemic juvenile idiopathic arthritis, polyarticular juvenile idiopathic arthritis, and cytokine release syndrome following chimeric cntigen ceceptor (CAR) T-cell therapy for acute lymphoblastic leukemia. There is substantial experience with this well-tolerated medication in the pediatric oncology community. Adverse events associated with tocilizumab are infection, neutropenia, thrombocytopenia, and elevated liver enzymes. This report discusses the management of two patients with cystic ACP who failed first line cystic-directed therapies and were eventually offered systemic administration of the IL-6R antibody, tocilizumab, on a compassionate use basis.

Primary patient samples were obtained from Children's Hospital Colorado and collected in accordance with local and Federal human research protection guidelines and institutional review board regulations. The protocol was approved by the Colorado Multiple Institutional Review Board (COMIRB 95–500). Written informed consent was obtained for all specimens and clinical information collected.

## Case 1 Presentation

**Figure 1:**
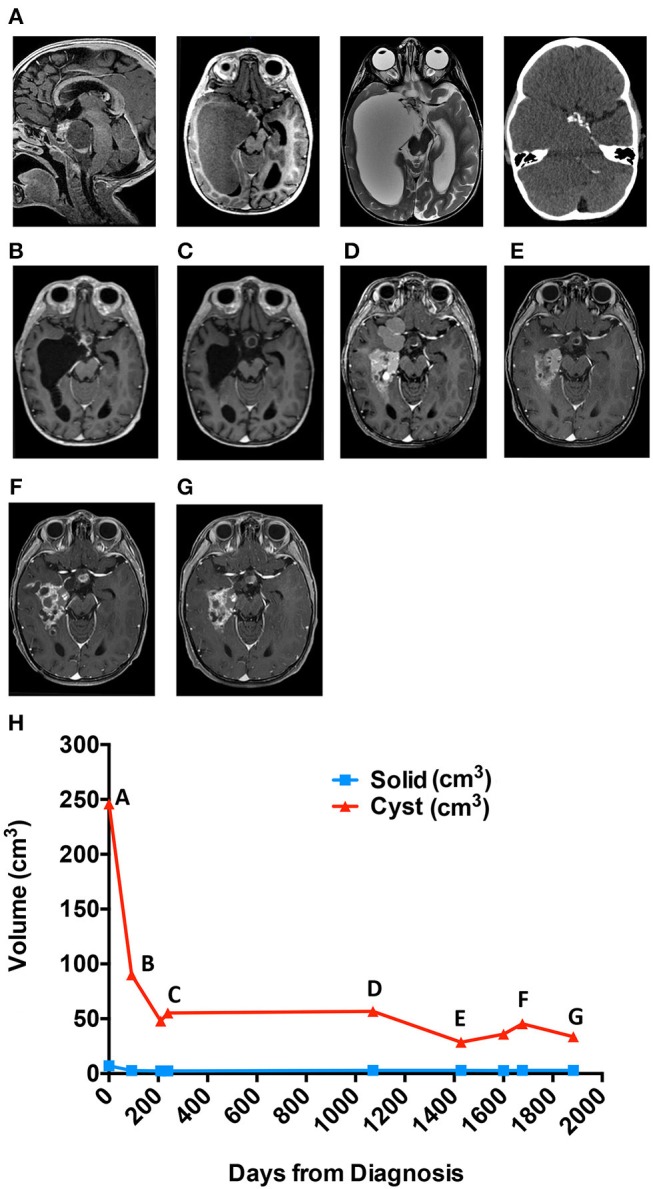
Cyst volume (cm^3^) in response to treatment course for patient 1. **(A)** Diagnostic MRis. **(B)** Early response to interferon. **(C)** Response at end of bleomycin. **(D)** Best response to tocilizumab alone. **(E)** Best response to tocilizumab and bevacizumab. **(F)** Six months off all therapy. **(G)** Most recent scan. **(H)** Cyst volume (cm^3^) in response to treatment course.

A 3-year-old male presented to the Emergency Department after hitting his head during a fall from a crib. CT scan revealed a suprasellar mass with extensive cysts extending throughout the right middle and bilateral posterior cranial fossae, as well as to the atrium of the right lateral ventricle. MRI (Figure 1A) confirmed the findings, which were most consistent with the diagnosis of craniopharyngioma. MRI-based manual segmentation software Aquarius (iNtuition, TeraRecon, Forest City, CA) was used to measure tumor volumes showing primary cystic disease with minimal solid tumor component (Figure 1, graph). The patient's medical history included premature birth at 34 weeks. He demonstrated normal growth and development until 13 months of age when he experienced speech and motor regression. He regained some motor skills prior to his presentation but continued to experience mild to moderate speech delay.

A cyst catheter was placed into the dominant right temporal cyst. Three days after discharge, he developed significant facial and scalp swelling accompanied by vomiting. This swelling was ultimately attributed to cyst fluid tracking along the outside of the catheter and under the Ommaya reservoir into the subcutaneous soft tissues. He was successfully treated with a course of oral dexamethasone.

Approximately 6 weeks following placement, he was treated with intracystic IFN-α according to previously utilized protocols (11, 18). Overall cyst volume initially decreased (Figure 1B) however, after 4 months of therapy, the cystic component began to increase in size. The patient was transitioned to intracystic bleomycin therapy and received 3 units/10 mls three times per week for a total of 14 doses.

Following 5 weeks of intracystic bleomycin, he presented emergently with new onset left VI and VII cranial nerve palsies. MRI demonstrated new edema involving the left pons, middle cerebellar peduncle, and anterior right cerebellar hemisphere, in continuity with one wall of the cyst receiving bleomycin (Figure 1C). Bleomycin therapy was discontinued, and the patient was treated with dexamethasone, resulting in full recovery of CN VI and partial recovery of CN VII function, with residual mild facial asymmetry and weakness.

The patient remained off of therapy with routine MRI scanning to monitor for disease progression. During the initial 7 months, there was radiographic improvement of the cystic portions of his lesion however, imaging at 14 months off therapy demonstrated progression of his cystic disease (Figure 1, graph).

Given his young age and the large potential radiation field, the risks of radiation therapy were thought to outweigh the benefit, and he was offered treatment with tociliziumab (12 mg/kg IV q2 weeks) on a compassionate use basis, which he received for 6 months and tolerated well. Imaging 6 months following the start of therapy demonstrated a decrease in volume of some cysts and an apparent slowing of expansion of others (Figure 1D). Following an additional 3 months of therapy, the patient was admitted to the Emergency Department for seizure episodes coincident with the collapse of multiple cysts, although it could not be definitively determined that the seizures were related to the cyst collapse. He underwent a complete workup for evaluation of his seizures and was found to have normal metabolic labs, no evidence of vascular changes, and no residual edema from his previous bleomycin treatment. EEG evidence shows seizures arise in the right temporal region which is associated with significant cystic disease burden and felt to be the underlying cause of his seizures. His seizures were controlled with levetiracetam and he continued treatment with tocilizumab. Following 8 months of therapy, MRI showed evidence of progression of the cystic component of his ACP (Figure 1, graph). The patient experienced increased headaches and required intensified supportive care including narcotic pain medication to support his pain control needs. Treatment options were discussed including surgery and radiation, but both options were deemed to have significant morbidity risks by the family, and they requested an alternative chemotherapy plan. At this time, an attempt to treat his disease with combination bevacizumab and tocilizumab was initiated. Bevacizumab was added as potential additional cyst directed therapy.

Systemic IV tocilizumab in combination with IV bevacizumab every 2 weeks was generally well tolerated. There were three delays in therapy of 2 weeks for the first two delays and 4 weeks for the third delay due to neutropenia (CTCAE v5, Grade 3). There were no fevers or signs of systemic illness associated with these episodes. In total, he received 14 months of combination therapy over 28 months with MRIs showing a significant decrease in cystic disease (Figure 1E). Following multiple scans with stable disease, the primary oncologist and family elected for a therapy break with routine tumor surveillance.

Three months off of therapy, an MRI showed a minimal increase in some cystic components while the remainder of the solid and cystic components remained unchanged. Six months off therapy the patient was seen in the Emergency Department with seizure activity. At this time, an MRI demonstrated an overall increase in his cystic mass (Figure 1F). Bevacizumab and tocilizumab were re-initiated with a significant decrease in cystic disease following 4 months of therapy. The patient has remained on combination therapy for 13 months (Figure 1G), to date, and his seizures are controlled with levetiracetam.

## Case 2 Presentation

A 7-year-old male presented with a 1 month-long history of nausea and vomiting. A head CT was performed for concern for increased intracranial pressure and demonstrated a large cystic frontal mass that crossed the midline, with perilesional edema. His past medical history was significant for a diagnosis of “lazy eye” 1 year previously and short stature (later determined to be due to growth hormone deficiency). Family history was unremarkable.

**Figure 2:**
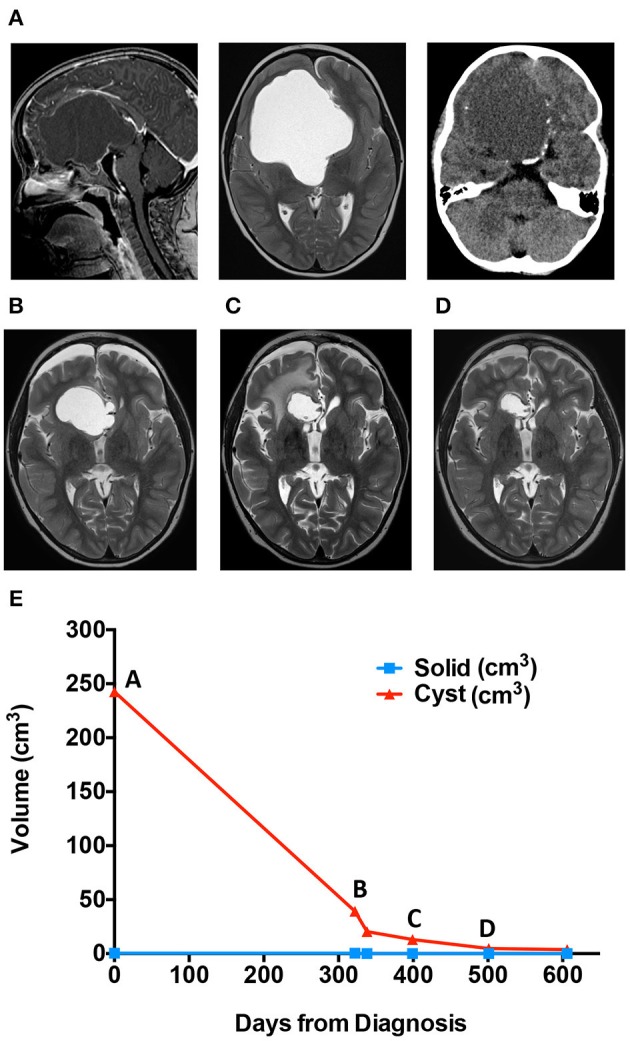
Cyst volume (cm^3^) in response to treatment course for patient 2. **(A)** Diagnostic MRis. **(B)** Scan immediately before ommaya reservoir replacement. **(C)** Scan immediately stopping bleomycin for right frontal lobe edema. **(D)** Scan while on Tocilizumab. **(E)** Patient two cyst volume (cm^3^) in response to treatment course.

MRI brain demonstrated a large cystic sellar/suprasellar mass, most consistent with craniopharyngioma (Figure 2A). MRI volume measures as with Case 1 demonstrated primary cystic disease with minimal solid tumor component (Figure 2, graph). Initial surgical management included stereotactic cyst catheter placement with concomitant drainage of ~50 mL of “machine oil”-like cyst fluid. His initial post-operative course was uneventful.

The patient returned to the outpatient clinic for weekly cyst aspiration. Three months after catheter placement, MRI demonstrated an increase in cyst size (Figure 2B), and repositioning of the catheter was undertaken. Resumption of scheduled cyst aspiration again led to a decrease in the cyst size (Figure 2, graph). However, due to the need for continued cyst aspiration, and the position of the tumor, which appeared amenable to gross surgical excision, craniotomy for tumor removal was undertaken. At surgery, the cyst wall was densely adherent to the anterior cerebral vessels, and subtotal resection was achieved. The patient was treated with focal radiation (54 Gy in 30 fractions) but was noted to have rapid cyst recurrence during radiation. Following completion of radiation, a cyst catheter was replaced. The patient was treated with intracystic bleomycin, which resulted in an initial decrease in cyst size. However, after 1 month, the patient developed new onset lethargy, and imaging demonstrated right frontal lobe edema adjacent to the cyst wall (Figure 2C). He was treated with dexamethasone and cessation of bleomycin with resolution of his symptoms.

The patient was offered systemic tocilizumab (12 mg/kg IV q2 weeks) on a compassionate use basis. After 6 months of therapy, the cyst exhibited a partial response with measurements improved from 26 × 28 × 27 mm (AP × TRV × CC) to 18 × 24 × 18 mm (AP × TRV × CC) (Figure 2D). He has had no significant adverse effects, no new neurologic sequela and no hospitalizations. Therapy was discontinued after seven months, and he continues on an MRI monitoring plan.

## Discussion

**Figure 3:**
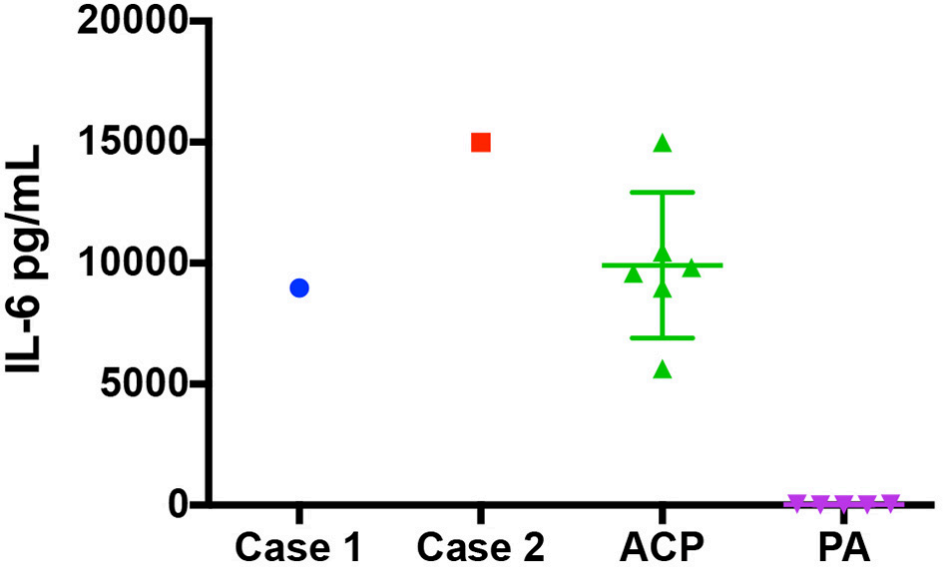
IL-6 Levels at diagnosis for patient 1, patient 2, a group of ACP Patients, and a group of pilocytic astrocytoma. The IL-6 levels (pg/mL) found in the cystic fluid of patient 1 and patient 2 at diagnosis as well as a group of 6 craniopharyngioma patients and a group of 5 patients with pilocytic astrocytoma.

Here we report the first use of tocilizumab alone or in combination with bevacizumab for the treatment of cystic ACP in pediatric patients. The biological basis for this was based on recent studies that demonstrated elevated IL-6R levels and IL-6 transcript levels in ACP cyst fluid and solid tissue, respectively. The patient in case 1 had known elevated IL-6 in cyst fluid prior to therapy with tocilizumab and level were in line with other samples tested (Figure 3). Case 2 had confirmation of elevated IL-6 in pre-therapy cyst fluid after initiation of therapy (16, 17). In addition, genetically engineered mouse models of ACP induced by mutation in anterior pituitary progenitors faithfully recapitulate many histological and biological characteristics of human ACP (15, 19) implying that the tumor may arise from tissue that lacks blood-brain barrier protection. Thus, systemically administered therapy may achieve sufficient tissue levels at the site of the lesion. In both cases presented, the cystic portion of tumor responded, further supporting the potential relevance of this therapy in children with cystic ACP.

Current therapies for pediatric ACP can cause significant morbidity, and safer, more effective treatment is needed (20). Research is expanding these options to include a number of biologic approaches. This includes the potential to target beta-catenin (15, 16, 21) and the use of MEK inhibitors (19). Here we propose new potential agents, tocilizumab alone or in combination with bevacizumab. Since Patient 1 progressed on tocilizumab alone, bevacizumab was added as a cyst targeted therapy. Bevacizumab, a monoclonal antibody that binds to vascular endothelial growth factor (VEGF) resulting in inhibition of new blood vessel formation. No research has been done on the use of bevacizumab in ACP patients, but one small case series reviewed the use of bevacizumab in three patients with pilocytic astrocytoma (PA), another predominantly cystic tumor, and found that all three patients tolerated the therapy well and resulted in a decrease cyst volumes (22). Bevacizumab has also been shown recently to have clinical and radiographic effects in adult PA patients (23, 24) and has been shown to be a safe and tolerated therapy in pediatric patients with PA and other forms of low-grade glioma (25, 26). Overall, the patients presented tolerated therapy well without significant side effects or complications other than isolated Grade 3 neutropenia without associated infections.

These two case studies suggest that the combination use of systemically administered tocilizumab and bevacizumab may be effective in pediatric patients with primarily cystic craniopharyngioma. Future evaluation of this combination of this drug is needed to further evaluate the potential of these drugs in the management of ACP.

## Author Contributions

SG did primary case reviews and writing of original draft of manuscript. DM provided MRI analysis and figures. AD and NF provided laboratory data. ND and LH provided patient care and manuscript preparation. TH and JM provided primary patient care, conceptualization, manuscript preparation, and project management.

### Conflict of Interest Statement

The authors declare that the research was conducted in the absence of any commercial or financial relationships that could be construed as a potential conflict of interest.
